# The present and future use of functional near‐infrared spectroscopy (fNIRS) for cognitive neuroscience

**DOI:** 10.1111/nyas.13948

**Published:** 2018-08-07

**Authors:** Paola Pinti, Ilias Tachtsidis, Antonia Hamilton, Joy Hirsch, Clarisse Aichelburg, Sam Gilbert, Paul W. Burgess

**Affiliations:** ^1^ Department of Medical Physics and Biomedical Engineering University College London London UK; ^2^ Institute of Cognitive Neuroscience University College London London UK; ^3^ Department of Psychiatry Yale School of Medicine New Haven Connecticut; ^4^ Department of Neuroscience Yale School of Medicine New Haven Connecticut; ^5^ Comparative Medicine Yale School of Medicine New Haven Connecticut

**Keywords:** fNIRS, basics of fNIRS, cognitive neuroscience, social neuroscience, ecological

## Abstract

The past few decades have seen a rapid increase in the use of functional near‐infrared spectroscopy (fNIRS) in cognitive neuroscience. This fast growth is due to the several advances that fNIRS offers over the other neuroimaging modalities such as functional magnetic resonance imaging and electroencephalography/magnetoencephalography. In particular, fNIRS is harmless, tolerant to bodily movements, and highly portable, being suitable for all possible participant populations, from newborns to the elderly and experimental settings, both inside and outside the laboratory. In this review we aim to provide a comprehensive and state‐of‐the‐art review of fNIRS basics, technical developments, and applications. In particular, we discuss some of the open challenges and the potential of fNIRS for cognitive neuroscience research, with a particular focus on neuroimaging in naturalistic environments and social cognitive neuroscience.

## Graphical abstract

The past few decades have seen a rapid increase in the use of functional near‐infrared spectroscopy (fNIRS) in cognitive neuroscience. In our review, we aim to provide a comprehensive and state‐of‐the‐art review of fNIRS basics, technical developments, and applications. In particular, we discuss some of the open challenges and the potential of fNIRS for cognitive neuroscience research, with a particular focus on neuroimaging in naturalistic environments and social cognitive neuroscience.

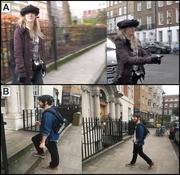

## Introduction

Advances in functional neuroimaging technologies and research have had a major impact on our understanding of a wide range of brain functions, not only in case of the healthy brain, but also in neurological and psychiatric conditions. However, it is still challenging to understand how performance in tightly controlled cognitive tasks relates to our everyday lives and can be of value in the diagnosis and treatment of clinical conditions. Some of these challenges arise from the constraints of neuroimaging technologies themselves. The present review article describes an emerging technology—functional near‐infrared spectroscopy (fNIRS)—which has the potential to overcome some of these issues.

The past 25 years have seen rapid growth of fNIRS as a tool to monitor functional brain activity in a wide range of applications and populations. One of the most successful areas of investigation with fNIRS has been neurodevelopment.^1^ Some of the attributes of fNIRS such as portability, movement tolerability, and safety of use have made this technique particularly suitable for investigating brain function in infants and children. As recently reviewed in Ref. 2, fNIRS shed light on several aspects of cognition in the developing brain that could not be properly studied with other neuroimaging modalities such as functional magnetic resonance imaging (fMRI), including object, face and language processing, and functional specialization within the visual, auditory, and sensorimotor systems. Additionally, exciting research has been carried out in the study of atypical development, with a focus on executive and language dysfunctions, and neurodevelopmental disorders such as autism and attention‐deficit hyperactivity disorder that are difficult to investigate in tightly restrained environments as, for example, an fMRI scanner. In particular, the functional organization and the social brain within autism have been investigated both in children^3^, ^4^ and adults,^5^ showing the ability of fNIRS to measure neurocognitive functions in these groups.

fNIRS has also been demonstrated to be a very promising tool to investigate cortical perturbations in psychiatric conditions (see Ref. 6 for a review). For instance, substantial research has been conducted into schizophrenia research, particularly looking at functional abnormalities within the prefrontal cortex (PFC) during verbal fluency tasks. In general, differences in hemodynamic response dynamics,^6^ reduced brain activity,^7^ and atypical functional connectivity patterns^8^ were observed in individuals with schizophrenia.

Low sensitivity to body movements and the systems’ portability make fNIRS suitable for monitoring cortical hemodynamics during motor tasks or during tasks involving walking, which is not fully possible in the restrained environment of scanners. This is useful to map functional activation patterns during everyday life activities (e.g., investigate the increase in the risk of falling when using the smartphone while walking^9^ or investigate mental workload during navigation^10^) and to explore the effects of neurorehabilitation.^11^ For example, the work in Ref. 12 evaluated the changes in cortical activation in stroke patients before and after 2 months of rehabilitation. This study demonstrated the potential of fNIRS in detecting changes in regional activation associated with locomotor recovery, as reflected by an increase in the activation of premotor cortex in the affected hemisphere after rehabilitation. Other studies using wearable fNIRS investigated the contribution of dysfunctions within PFC to difficulties in performing a second task while walking in different disorders (e.g., Parkinson's disease,^13^ elderly people with mild cognitive impairment^14^, ^15^). In this way, fNIRS has shown clear potential as a neurorehabilitation tool to monitor the motor and cognitive progresses of patients over time. This is in addition to its use as a communication device in the brain–computer interface systems for people with motor disabilities.^16^

Our review builds upon the exciting work carried out over the past few decades that have established fNIRS as a stable and reliable neuroimaging methodology to discuss new applications in those fields of cognitive neuroscience that would benefit the most from the use of this technology. In particular, we first summarize the history of fNIRS and how it works, before providing a more detailed comparison with other neuroimaging methods. We then move on to explore how new studies use fNIRS in the domains of executive function and social cognition, and highlight the scope for exciting future developments.

## History and basics of near‐infrared (NIR) spectroscopy

fNIRS is an optical, noninvasive neuroimaging technique that allows the measurement of brain tissue concentration changes of oxygenated (HbO_2_) and deoxygenated (HbR) hemoglobin following neuronal activation. This is achieved by shining NIR light (650–950 nm) into the head, and, taking advantage of the relative transparency of the biological tissue within this NIR optical window, light will reach the brain tissue. For further information about the fNIRS technology, see recent review in Ref. 17.

The discovery of the existence of the NIR optical window into our body dates back to 1977,^18^ when Fransis Jöbsis observed the capability of red light to penetrate through a 4‐mm‐thick bone of a beefsteak while he was holding it against visible light.^19^ This suggested that red light and even more NIR light with longer wavelengths, could travel through our scalp and skull and reach the underlying tissues. The fNIRS technology takes advantage of the transparency property of the skin and bones to NIR light, and has been used in many different fields and applications, including the investigation of muscle physiology (see Ref. 20) and the clinical monitoring of cerebral cortex pathophysiology (see Ref. 21 for a review). For a detailed review of the history of the development of fNIRS, we advise the reader to see Ref. 22. Briefly, in the early 1990s, fNIRS recordings were initially performed using single‐site (or single‐channel) devices and demonstrated the capability of fNIRS to measure oxygenation and hemodynamic changes in the brain in response to functional activation tasks both in adults (e.g., mental arithmetic tasks over the left PFC,^23^, ^24^ visual stimulation over the occipital cortex (OC),^24^, ^25^, ^26^ and analogy task^27^) and infants (e.g., visual stimulation over the OC^28^). To fully exploit and expand the potentiality of fNIRS, multisite (or multichannel) measurements were needed.^29^ Initially, single‐site devices were combined and simultaneously used at multiple locations;^30^ later, the first multichannel systems were developed, allowing monitoring of larger portions of the head and the gathering of topographic HbO_2_ and HbR maps.^29^ Further proof‐of‐principle studies were thus conducted, exploring the brain hemodynamic changes to basic functional demands at more locations simultaneously (e.g., finger tapping tasks,^31^, ^32^ verbal fluency task^33^) and validating fNIRS as a reliable functional neuroimaging tool.^34^

As more sophisticated multichannel and wearable instrumentation have been developed and put to use in cognitive experiments, fNIRS has led to important advances in the understanding of functional brain activity and higher cognitive functions in adults and babies, both in health and disease (see Refs. 17, 21, 35, and 36). Growth in the fNIRS community is now exponential,^21^ with the number of papers published in journals doubling every 3.5 years.^37^ For instance, *NeuroImage*, a highly relevant journal for the neuroimaging community, dedicated a special issue to commemorate the first 20 years of fNIRS research in 2014.^37^ The success of fNIRS as a neuroimaging tool led to the constitution of the Society for fNIRS^38^ in 2014 that aims to bring together the fNIRS community to collaborate and strengthen our knowledge on use of fNIRS for understanding the properties and functioning of the human brain. The Society holds the official fNIRS conference every 2 years, with an increasing number of submission (from 49 submissions in 2010, to 221 in 2014, and 247 in 2016^38^, ^39^), and recently adopted *Neurophotonics* as its official journal, to further foster the strong relationship between optics and neuroscience.

### Physical principles of fNIRS

fNIRS measurements are carried out by transmitting NIR light onto the scalp. Prior to reaching the brain, the NIR light has to travel through several different layers (e.g., the scalp skin, skull, cerebrospinal fluid), each with different optical properties. The interaction of the NIR light with the human tissue is thus complicated as the tissue is anisotropic and inhomogeneous through the different layers. However, this can be simplified considering that the NIR light is attenuated by absorption and scattering.^40^

Absorption is the process by which the energy of a photon is converted into internal energy of the medium it is travelling in, and depends on the molecular properties of the material. In our tissue, there are several substances, such as water, lipids, hemoglobin, melanin, and cytochrome‐c‐oxidase, each with different absorbing properties at the different wavelengths.^17^ In particular, our body is made of approximately 70% of water and, in the NIR optical window, its absorption is minimum, allowing the NIR light to travel through the tissue. The most dominant and physiological‐dependent absorbing chromophore within the NIR optical window is hemoglobin. Based on its saturation state, we can have hemoglobin in its oxygenated (i.e., oxyhemoglobin, HbO_2_) and deoxygenated form (i.e., deoxyhemoglobin, HbR). In particular, HbO_2_ and HbR absorb the NIR light differently: HbO_2_ absorption is higher for λ > 800 nm; on the contrary, HbR absorption coefficient is higher for λ < 800 nm. This difference in absorption reflects also on the color of the blood that is more red for oxygenated blood (arterial blood, ≈98% saturated) and more purple for venous blood (≈75% saturated), and can be quantified through spectroscopic measurements.

**Figure 1:**
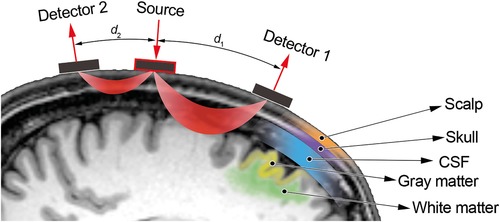
Illustration of the path (shown in red) followed by the NIR photons from the light source to the detector through the different layers of the head. The penetration depth of the light is proportional to the source–detector distance (*d*_1_: deeper channel; *d*_2_: superficial channel). A channel is composed by the pair source–detector and is located at the midpoint between the source and the detector and at a depth of around the half of the source–detector separation.

When a brain area is active and involved in the execution of a certain task, the brain's metabolic demand for oxygen and glucose increases, leading to an oversupply in regional cerebral blood flow (CBF) to meet the increased metabolic demand of the brain. The increase in CBF in response to an increase in neuronal activity is called functional hyperemia and is mediated by several neurovascular coupling^41^ mechanisms, such as changes in capillary diameter and vasoactive metabolites.^42^ Hence, the oversupply in regional CBF produces an increase in HbO_2_ and a decrease in HbR concentrations; these are estimated by changes in light attenuation that can be measured by fNIRS. In addition to absorption, the NIR light is also scattered when it travels through the biological tissue. Scattering is 100 times more frequent than absorption and leads to light attenuation. The more a photon is scattered, the longer is the travelled path and the greater is the probability of being absorbed. The light shined into the head will be scattered, diffused, and able to penetrate several centimeters through the tissue (Fig. 1).

**Figure 2:**
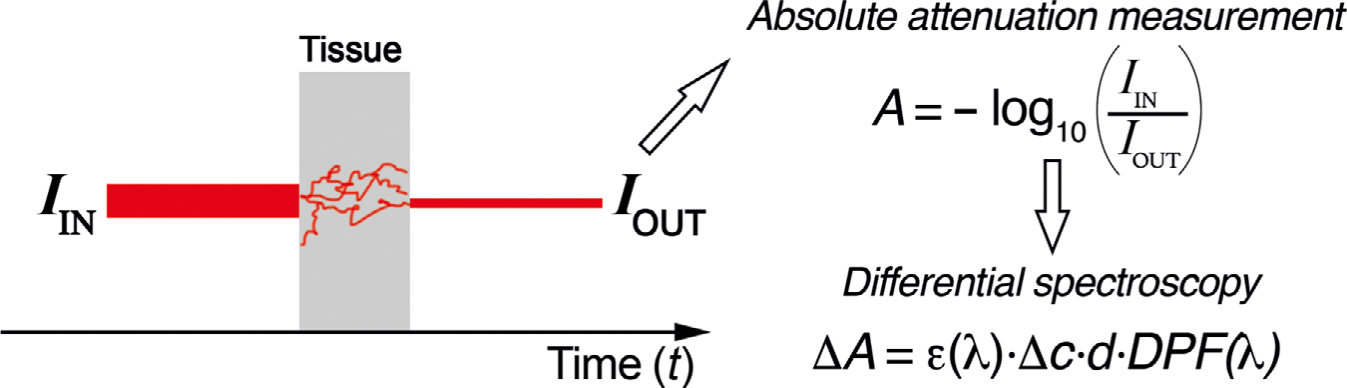
Continuous wave devices measure light attenuation due to scattering and absorption based on intensity measurements of the input (*I*_IN_) and output (*I*_OUT_) light. Attenuation is evaluated by computing the logarithm of the ratio between *I*_IN_ and *I*_OUT_ that is related to changes in hemoglobin concentration. The first attenuation measurement is subtracted to the following attenuation values to remove the effect of scattering, melanin, and water concentrations (differential spectroscopy). The changes in attenuation Δ*A* are related to the changes in chromophore concentrations (Δ*c*, either HbO_2_ or HbR) by the modified Beer–Lambert law. Note that *d* represents the source–detector distance, *ε* is the extinction coefficient of the chromophore at a certain wavelength *λ*, and the *DPF* is the differential pathlength and indicates the increase in the photon path due to scattering.

Therefore, if we place a light detector at a certain distance from the NIR light source, we are able to collect the backscattered light (Fig. 1) and to measure changes in light attenuation. As absorption within the NIR optical window is mainly due to HbO_2_ and HbR, changes in light attenuation at a given wavelength can be expressed as a linear combination of concentration changes of HbO_2_ and HbR. Most of the commercially available systems, known as continuous wave (CW) fNIRS instruments, use continuously emitted NIR light, typically at two or three wavelengths, and measure light attenuation (*A*) due to tissue scattering and absorption through estimating the ratio of the injected (*I*_IN_) to the output (*I*_OUT_) light as shown in Figure 2. By subtracting the first attenuation measurement from the following attenuation measures, changes in attenuation (Δ*A*) are estimated and used to derive the changes in concentration of HbO_2_ and HbR. This assumes that Δ*A* is only dependent on the changes in absorption by the oxygen‐dependent hemoglobin chromophores, therefore removing other factors such as scattering, melanin, and water concentrations, which are unlikely to change significantly during the measurement period. This method is often referred to as the modified Beer–Lambert law or differential spectroscopy and is widely applied in fNIRS.^40^

CW fNIRS devices can provide information on concentration changes of HbO_2_ and HbR but cannot resolve absolute baseline concentrations, as they are not able to separate and quantify the contribution of absorption and scattering. That is why measurements of HbO_2_ and HbR start from zero. However, these systems are well suited for applications in cognitive neuroscience as absolute concentrations are not essential and functional activity is usually evaluated relatively to a baseline.

Besides the fNIRS systems based on the CW technology, fNIRS instruments can also be divided in other two classes: time‐domain (TD) and frequency‐domain (FD) devices. These allow the light absorption and scattering contributions to be separated, thus obtaining absolute HbO_2_ and HbR concentrations. FD devices shine the brain with intensity‐modulated NIR light, while TD systems are more sophisticated and implement a NIR light source of few picosecond pulses and a fast time‐resolved detector to recover the time of flight (temporal spread function) of the re‐emerging photons. The temporal spread function provides information on the scattered and absorbed light but also on the depth reached by the photons within the brain (i.e., the higher the time spent inside the brain by the photons, the bigger the distance they reached). For further details on other types of fNIRS instrumentation, see Ref. 17.

The portion of tissue interrogated by the NIR light is called a *channel* and is located at the midpoint between the source and the detector, and at a depth of around the half of the source–detector separation.^43^ The penetration depth of the light is related to the source–detector distance (i.e., the longer the source–detector distance, the deeper the penetration). Several studies assessed the spatial and depth sensitivity of fNIRS to brain tissue as a function of different source–detector separations using Monte Carlo simulations. For instance, the investigation included in Ref. 44 suggested that a higher sensitivity to the brain tissue in adults can be obtained using a source–detector separation of 55 mm. However, increasing the source–detector separation to reach deeper structures leads to a signal‐to‐noise ratio (SNR) deterioration, as the probability of light absorption increases and less light is received by the detector.^45^ The source–detector separation thus has to be a compromise between depth sensitivity and SNR. Typical values that ensure this trade‐off are source–detector separations of 30–35 mm for adult studies and 20–25 mm for infants.

Generally, the fNIRS technology can be used in two ways, based on the number of channels and their configuration. In its simple, most common and commercially available form, fNIRS source and detector optical fibers (or optodes) are distributed uniquely at various locations on the head and at fixed source–detector separations. Each source–detector separation represents a measuring channel providing topographical representation of the distribution of the changes in concentration of HbO_2_ and HbR over the cortical surface. An alternative configuration requires the use of overlapping channels by applying multiple source–detector distances over the head to acquire tomographical representation of the distribution of the changes in concentration of HbO_2_ and HbR over the cortical surface. This latter configuration of fNIRS is referred to as diffuse optical tomography (DOT), where much denser arrays of channels are used that sample overlapping brain volumes.^46^

More recently, wearable and/or fibreless fNIRS instruments were developed. These devices are based on the CW technology, battery‐powered, and usually use LEDs directly coupled to the head. The absence of fiber optics bundles makes them more lightweight and more robust to movement artifacts. Participants can thus move more naturally with fewer constraints. Data can be usually recorded on the wearable recording unit or sent wirelessly to a laptop.

### fNIRS and the hemodynamic response

**Figure 3:**
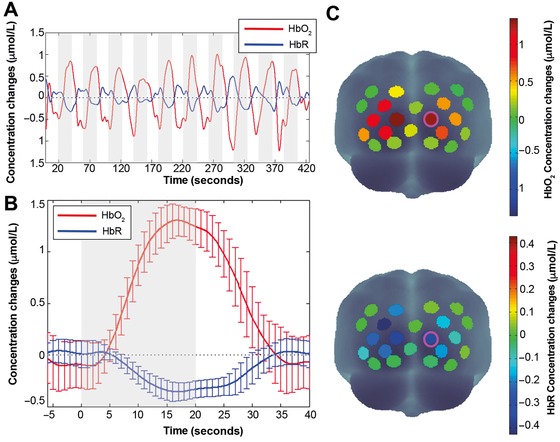
Example of HbO_2_ (red) and HbR (blue) signals from a representative channel (circled in magenta in panel C) of a single subject over the visual cortex using the fNIRS Hitachi ETG‐4000 (equipped with up to 52 channels) during a block‐designed flashing checkerboard experiment, stimulating the occipital cortex bilaterally (A). The gray areas refer to the stimulation period. Panel B shows the block‐averaged hemodynamic response (mean ± SD) computed by averaging the HbO_2_ and HbR signals presented in A across the 10 task blocks. It is characterized by simultaneous HbO_2_ increase and HbR decrease. Panel C presents the distribution of the maximum block‐averaged concentration changes within the gray block shown in Panel B across all the channels, both for HbO_2_ (top) and HbR (bottom). The bilateral occipital cortices consistently respond to the full flashing checkerboard, as shown by the more red for HbO_2_ and more blue HbR channels.

Neural activity is associated with an increase in local arteriolar vasodilation and the subsequent oversupply in CBF and increase in cerebral blood volume (CBV), that is, functional hyperemia,^42^ to support the increase in neuronal demand for nutrients (i.e., glucose and oxygen). The amount of oxygen that reaches the activated brain region is higher than the rate at which it is consumed, leading to an increase in HbO_2_ and decrease in HbR (Fig. 3A). This is called a *hemodynamic response* and can be measured through fNIRS (Fig. 3B) at multiple locations of the cerebral cortex (Fig. 3C). Data in Figure 3 refer to a visual stimulation with a flashing checkerboard.

**Figure 4:**
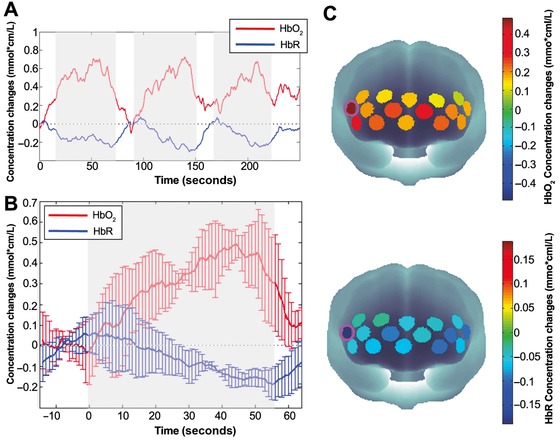
Example of HbO_2_ (red) and HbR (blue) signals from one channel (circled in magenta in panel C) of a single subject over the PFC using the fNIRS Hitachi WOT system (equipped with 16 channels) during a block‐designed prospective memory experiment (A). The gray areas refer to the stimulation period. Panel B shows the block‐averaged hemodynamic response (mean ± SD) computed by averaging the HbO_2_ and HbR signals presented in A across the task blocks. It is characterized by simultaneous HbO_2_ increase and HbR decrease. Panel C presents the distribution of the maximum block‐averaged concentration changes within the gray block shown in panel B across all the channels, both for HbO_2_ (top) and HbR (bottom). PFC activity was elicited by the prospective memory task, as shown by the more red for HbO_2_ and more blue for HbR channels.

The same is true for cognitive experiments that actively engage the participant in performing a functional task recruiting high‐level cognitive functions, as show in Figure 4. Data refer to a prospective memory task where participants were presented with two pictures of objects and had to judge which one was heavier while responding with a different keyboard button to a target object. fNIRS signals were recorded over the PFC and increase in HbO_2_ and decrease in HbR (Fig. 4A and B) can be observed at different locations of the measured brain region (Fig. 4C).

Generally during a stimulus event, the hemodynamic response reaches a peak at ∼5 s after the stimulus onset and goes back to its baseline with a certain delay (∼16 s from the stimulus onset).^47^ The response dynamics (e.g., peak and undershoot latency, duration) can vary across different brain regions, task types and design, and participants’ age.

**Figure 5:**
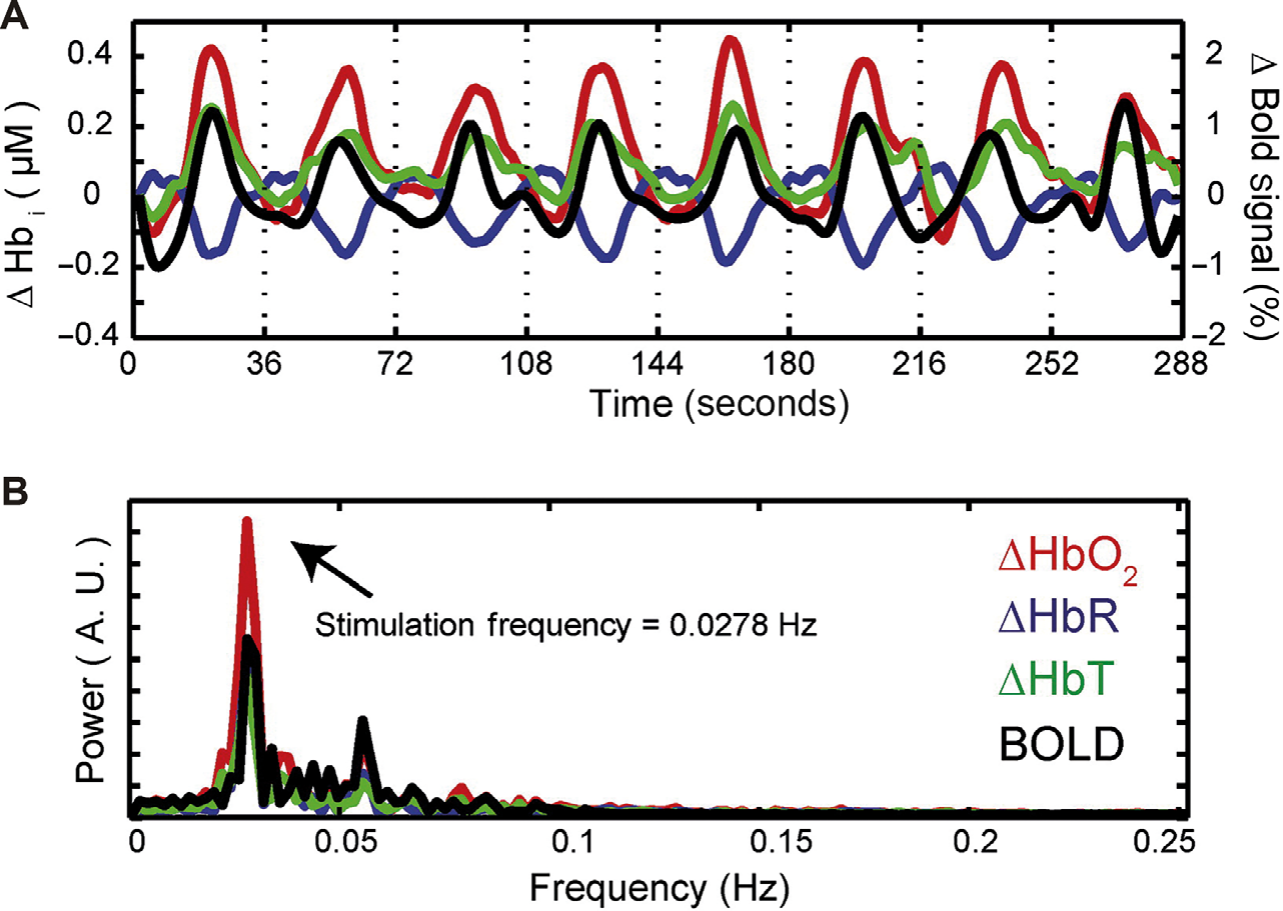
Example of HbO_2_, HbR, and HbT (HbT = HbO_2_ + HbR) signals measured with fNIRS and BOLD signal measured with fMRI in the occipital cortex during visual stimulation (A). Panel B shows the frequency spectra of the four signals. The figure is taken from the study in Ref. 51 using an in‐house developed diffuse optical tomography system equipped with 24 light sources and 28 detectors. The figure is reprinted from Ref. 51 with permission from Elsevier.

Several studies^48^, ^49^, ^50^, ^51^, ^52^ have been conducted to validate and to compare the metabolic correlates of neural activity as measured by fNIRS (i.e., increase in HbO_2_ and decrease in HbR) with the gold standard measured by fMRI (i.e., the blood oxygenation level–dependent (BOLD) response). Positive correlations between the BOLD signal and HbO_2_ were found as well as anticorrelations with HbR.^50^ Figure 5 shows an example of changes in HbO_2_, HbR, and total hemoglobin (HbT) compared to the BOLD signal during visual stimulation, both in the TD (Fig. 5A) and FD (Fig. 5B); data refer to the study in Ref. 51.

### Analysis of fNIRS data

Together with the progress in hardware development and novel applications of fNIRS, the analysis methods of fNIRS data evolved over the years as well (see Ref. 53 for a review). Initially task‐evoked brain activity was inferred using qualitative approaches (e.g., visually inspecting the signals or using amplitude thresholds). Now, more statistically meaningful and reliable methods are deployed. These include event‐locked or task‐locked averaging of specific portions of the continuous fNIRS signal; statistical tests can then be applied to the average activation for different events. Given the similarity in the hemodynamic signals measured by fNIRS and fMRI, more sophisticated analysis methods commonly used for fMRI have been expanded to the analysis of fNIRS data as well. A typical example would be the widely used statistical parametric mapping approach^54^ based on the General Linear Model (GLM). This method consists of fitting the fNIRS data with a linear combination of explanatory variables (i.e., regressors) that reflect the stimulation protocol design. The GLM thus has more statistical power than averaging as it considers the entire fNIRS time series and takes advantage of the higher sampling rate (commonly up to 10 Hz) of fNIRS recordings.^53^ Other data‐driven approaches detect functional activity assuming that the recorded fNIRS data are a combination of task‐evoked and task‐independent components that can be separated by means of independent component analysis (ICA),^55^ principal component analysis (PCA),^56^ or task‐related component analysis.^57^ Recently, more powerful and multivariate techniques, such as the multivoxel pattern analysis (MVPA), have been borrowed from fMRI, with the capacity to discriminate task‐evoked brain activity between two or more experimental conditions.^58^ Freeware analysis toolboxes have been developed and listed on the Society for fNIRS.^59^

**Figure 6:**
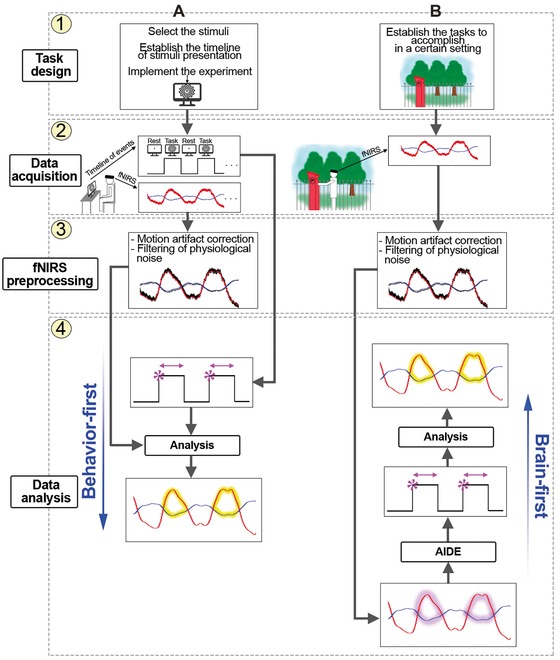
Example of neuroimaging experiment pipelines with fNIRS in case of typical computer‐based (A) and ecological (B) experiments. In the first case, the timeline of the stimuli is predetermined (A1) and fNIRS data are recorded synchronously to that (A2). Preprocessed fNIRS data (A3) are used to assess the presence of significant hemodynamic changes (yellow areas, A4) feeding conventional analysis methods (A4) with the events timeline (behavior‐first). In ecological experiments, tasks do not have a particular structure (B1) and fNIRS data are recorded continuously (B2). New methods such as AIDE (B4) are then able to recover the timeline of functional events from preprocessed fNIRS data (B3, brain‐first) by looking at particular patterns in HbO_2_ and HbR signals (magenta areas, B4). The recovered events can be used to assess the presence of functional activation (yellow areas, B4).

All these methods rely on knowledge of the timeline of the stimuli that is predetermined and recorded in typical computer‐based experiments conducted in laboratories. An example of a computer‐based fNIRS experiment pipeline is shown in Figure 6A. Typically, the sequence of the chosen stimuli is established and coded into a presentation software (Fig. 6A1) that can be also used to trigger the fNIRS recording. During the data acquisition step (Fig. 6A2), stimuli are presented to the participant, usually following a block‐design or event‐related design structure (i.e., the stimulus is repeated several times alternated to rest periods). fNIRS data are recorded synchronously to the stimuli presentation and the timeline of the events is stored as well. fNIRS data are then preprocessed (Fig. 6A3) to remove errors due to head motions and physiological interferences, and used to infer functional brain activity (Fig. 6A4). Conventional analysis methods (e.g., averaging or GLM) start from the recorded timeline of the events to search for significant changes in HbO_2_ and HbR within the recorded task time period (yellow areas, Fig. 6A4). This can be called a *behavior‐first* approach as it is based on linking the occurrence of neural events to behaviors taking place at a certain time (e.g., watching a flashing checkerboard on a computer screen or responding to a Stroop task).

However, the timeline of the stimuli is not always known for experiments performed in real‐world contexts where participants have a free choice of where to move and what to engage with. As an example, we focus on the study in Ref. 60 in which the PFC was monitored while participants completed a prospective memory task in a typical London street. Important events in this task occur at times determined by the participant (e.g., approaching a target parking meter, Fig. 6B1) or at times determined by the external world (e.g., a car driving by) and are not tightly controlled by the experimenters. One analysis approach is to video the whole task and then code the video footage to identify key events and make predictions about the timing of hemodynamic responses to those events, which can then be modeled in a GLM. However, this is very time consuming and may be inaccurate as it is difficult to estimate the exact moment when the participant encounters and processes a certain stimulus. To overcome this issue, other methods need to be adopted.

Recently, Pinti *et al*.^61^ developed such a method. They describe a new technique for the identification of the onsets of functional events directly from fNIRS data (Fig. 6B4). This algorithm, called AIDE (Automatic IDentification of Functional Events), works on preprocessed fNIRS data (Fig. 6B3) and takes the opposite approach, that is, a *brain‐first* (Fig. 6B4) one, since it looks at particular patterns (magenta areas, Fig. 6B4) in fNIRS data to detect the onsets and durations of functional events. The recovered events are then linked back to participants’ behavior and can be used to infer functional activation (yellow areas, Fig. 6B4). In this way, no *a priori* knowledge of stimuli presentation is needed.

### Measuring connectivity with fNIRS

Besides task‐evoked functional experiments, fNIRS is also widely used to assess resting‐state functional connectivity across brain regions. Functional connectivity concerns the investigation of the correlation in slow signal changes (<0.1 Hz) between different parts of the brain. The sampling rate of typical fNIRS systems is 10 Hz, which provides ideal data for connectivity measures with a reduced risk of aliasing of activity with higher frequency (e.g., heart rate (∼1 Hz)) into the lower frequencies (<0.1 Hz^62^), when compared to fMRI. Several studies showcase the flexibility of fNIRS for the assessment of cortical connectivity. The method has been validated using simultaneous fNIRS–fMRI measurements,^63^, ^64^ and is also suitable for infants^65^ with no need of sedatives.

In terms of analysis methods, classical approaches have been extended from fMRI to fNIRS such as the seed‐based correlation method^62^ that evaluates the correlation between a seed region and other regions. The seed can be an fNIRS channel and the correlation^66^ or the coherence at different frequency bands^67^ can be evaluated between all possible pairs of seed‐channels. However, the seed‐based approach strongly depends on the selection of the seed region and ignore the relation between multiple parts of the brain.^68^ To overcome this issue, other data‐driven approaches were proposed such as cluster analyses^62^ or ICA.^68^ Advanced fMRI methods including dynamic causal modeling have been applied to fNIRS data.^69^ Overall, fNIRS has exceptional potential in our growing understanding of brain connectivity, and may be particularly important in studying connectivity in development and in patient populations.

### Advantages and disadvantages of fNIRS

The utility of a particular neuroimaging technique can be assessed in a variety of ways, including the spatial and temporal resolution, the robustness of the data, potential sources of artifacts, potential for advanced analyses, and the accessibility of the method to a range of participants. Here, we review the advantages and disadvantages of fNIRS for neuroimaging investigations^70^ (Table 1), before providing a more focused comparison between fNIRS and other available neuroimaging modalities (Table 2).

**Table 1:** Advantages and disadvantages of fNIRS

Advantages	Disadvantages
Safe Better spatial resolution than EEG Better temporal resolution than fMRI Tolerance to motion artifacts Possibility to monitor HbO_2_ and HbR Portability Low cost Silent Availability of miniaturized and wearable systems Suitability for long‐time continuous monitoring Feasibility for multimodal imaging Compatibility with other electrical and magnetic devices More participant friendly than fMRI All participants are eligible (all ages, no exclusion criteria)	Lower temporal resolution than EEG/MEG Lower spatial resolution than fMRI Penetration depth (1.5–2 cm) Impossibility to gather structural images and anatomical information Systemic interferences Variable SNR Optodes placement can be time consuming in case of hairy regions and a high number of sources and detectors Higher susceptibility to motion errors and less comfort in case of high‐coverage measurements with fiber optics coupled to the head Lack of standardization in data analysis

**Table 2:** Comparison of fNIRS with other neuroimaging modalities

	fNIRS	fMRI	EEG/MEG	PET
Signal	HbO_2_ HbR	BOLD (HbR)	Electromagnetic	Cerebral blood flow Glucose metabolism
Spatial resolution	2–3 cm	0.3 mm voxels	5–9 cm	4 mm
Penetration depth	Brain cortex	Whole head	Brain cortex for EEG/deep structures for MEG	Whole head
Temporal sampling rates	Up to 10 Hz	1–3 Hz	>1000 Hz	<0.1 Hz
Range of possible tasks	Enormous	Limited	Limited	Limited
Robustness to motion	Very good	Limited	Limited	Limited
Range of possible participants	Everyone	Limited, can be challenging for children/patients	Everyone	Limited
Sounds	Silent	Very noisy	Silent	Silent
Portability	Yes, for portable systems	None	Yes, for portable EEG systems	None
Cost	Low	High	Low for EEG; high for MEG	High

fNIRS systems provide measurements from the cortical surface of two hemodynamic signals—HbO_2_ and HbR, as described above, with a spatial resolution of 2–3 cm. The investigation of the interrelationship between these two signals allows us to make more accurate conclusions about functional brain activity.^71^ Like fMRI, fNIRS records the hemodynamic response, which typically peaks after about 6 seconds. However, fNIRS systems have temporal sampling rates commonly up to 10 Hz, which massively oversamples the hemodynamic response function (HRF). This permits better tracking of the shape of an HRF.

One of the major factors driving the increase in fNIRS research is that this method has good tolerance to motion artifacts. A well‐positioned fNIRS cap will continue to give good signals when a participant walks (see Ref. 72 for a review), engages in conversation,^73^, ^74^ or even dances.^75^ New wearable fNIRS devices are becoming smaller, lighter and thus even more robust to bodily movements. A list of commercially available wearable fNIRS devices is provided in Ref. 70. This makes fNIRS suitable for a wider range of cognitive tasks, such as those requiring walking, for example, for neurorehabilitation purposes. In case of the optical displacement of the optodes (i.e., both NIRS light sources and detectors) due to rapid head movements, motion artifacts can appear as fast and narrow spikes or shifts from baseline values in fNIRS signals. Several techniques were developed to identify and effectively correct for motion errors,^76^ and were reviewed elsewhere.^77^ However, in case of high‐coverage or whole‐head measurements with fNIRS instruments guiding light through fiber optics, the weight of the probe holder increases, increasing the probability of having larger motion errors and corrupted signals.^70^

fNIRS systems have many advantages in cases where participant safety and comfort is a priority. fNIRS recording is silent, relatively comfortable and does not impose physical constraints on the participant, which means it is a method‐of‐choice for many studies of infants and children^35^ and for long recording sessions. Unlike fMRI, there are no safety concerns and fNIRS can be used with all participants from premature babies^78^ to patient populations (see Ref. 36 for a review).

To summarize, fNIRS can provide hemodynamic signals with high temporal sampling in a range of contexts and populations. This includes studies with freely moving participants,^60^ motor tasks,^79^ auditory stimuli,^80^ social neuroscience including hyperscanning of multiple participants simultaneously,^81^ clinical monitoring,^82^ and a range of participants including elderly people^83^ and infants.^35^ As the optical components do not interfere with electromagnetic fields, fNIRS is ideal for multimodal imaging (e.g., fNIRS–fMRI,^49^ fNIRS–EEG^84^) to gather more complete information related to neurovascular coupling and do not cause any harm when used on individuals with implanted therapeutic devices (e.g., cochlear implants).

Finally, it is worth mentioning that the influence of systemic blood‐flow changes on hemodynamic signals is a potential problem for both fMRI and fNIRS data, though it may be more pronounced in case of data recorded on freely moving people. The measured fNIRS signals are a combination of components arising from neuronal activity and components of systemic origin that can lead to false positives and/or false negatives in the statistical inference of functional activity. Tachtsidis and Scholkmann^71^ have discussed this issue and how to address it in a recent review.

Other available technologies for studying human cognition include fMRI and positron emission tomography (PET), which rely on neurovascular coupling, and electroencephalography (EEG) and magnetoencephalography (or MEG), which detect the electromagnetic activity of the brain. These are commonly compared based on the temporal and spatial resolution of each, but there are other important factors too. In particular, robustness to motion, and the breadth and variety of the participant sample that can be studied, are important factors in many contexts, especially where ecological validity is a priority. Table 2 summarizes the strengths and weakness of the neuroimaging technologies.

In general, however, it is important to highlight the fact that, although there are a wide range of fNIRS preprocessing and analysis procedures and free software available to the community, to date there is neither an agreement nor guidelines on the analysis of fNIRS data as in other well‐established technologies like fMRI. As pointed out by the recent article in Ref. 85, this lack of standardization and the numerous adjustable parameters for preprocessing and analysis algorithms can be confusing for new fNIRS users and can lead to poor quality studies or misinterpretation and irreproducibility of results. For instance, the authors^85^ demonstrated how different choices and combination of channels exclusion criteria, motion artifacts correction methods, signal denoising techniques, and inference approaches could lead to different results. In addition, a relevant issue in the fNIRS community concerns the investigation of which fNIRS‐derived signal can best be used to infer functional brain activity. fNIRS provides concentration changes of two signals, oxyhemoglobin and deoxyhemoglobin, each with its advantages and disadvantages. Theoretically, functional activation is reflected by concurrent increase in HbO_2_ and decrease in HbR. However, this is not always the case in real fNIRS data and, for example, only one chromophore might show significant changes in response to a certain task. This can happen for different reasons, for example, HbO_2_ changes are higher in amplitude and have a higher SNR but are more confounded by physiological interferences, while HbR can lack statistical power but is more robust to systemic changes.^71^ Therefore, conclusions in some papers are very often drawn based on only one chromophore, with no agreement on which one to use, and rarely on both. The hemoglobin species choice (HbO_2_, HbR, or combinations) thus also influences our conclusions, as demonstrated in Ref. 85. In summary, additional work and research are still required to establish guidelines and automated procedures for the analysis of fNIRS data to guarantee accurate inferences and localization of functional brain activity through fNIRS.

### Future directions in fNIRS hardware development

Significant improvements and advances have been made over the past two decades in terms of fNIRS instrumentation, recently leading to the development of wireless and wearable systems. We expect this progress to continue over the next years, with the extension and expansion of the current CW‐based devices to more advanced and sophisticated technologies that will push the boundaries of fNIRS applications and open the way to new applications. In particular, we highlight the developments of (1) DOT fNIRS instruments that can achieve imaging of cortical tissue with spatial resolution similar to fMRI. DOT can extend the current topographical representation of brain activity to three‐dimensional imaging and increase its spatial resolution through the combination of overlapping measurements at different depths and forward models of light propagation.^86^ However, in case of high‐density and whole‐head DOT with a very high number of optodes, the issues of weight reduction and optimization of the performance and ergonomics of such systems still need to be addressed;^87^ (2) TD fNIRS instruments that can perform depth resolved measurements and quantify light pathlength and light scattering, significantly improving the quantification of the hemoglobin concentrations.^88^ However, TD systems are more expensive than CW instruments and, to date the majority of them are prototypes developed in laboratories and not commercially available; (3) Broadband NIRS instruments that use hundreds of NIR wavelengths to measure changes in brain tissue cortical metabolism by quantification of the changes in the oxidation state of cytochrome C oxidase (or oxCCO). Most of these devices are CW based,^89^ but recently TD broadband instrumentation has also been developed.^90^ As before, prototype systems are currently used in some laboratories but they are not commercially available.

Great progress has been made so far in developing new DOT, TD, and broadband hardware. For instance, the cost and the size of TD devices were reduced by four orders of magnitude over the last 20 years.^88^ We expect even more advances that are exciting within the next 20 years, with miniaturized and less expensive fNIRS‐based instrumentation. Any future fNIRS instruments should be portable, easy to operate, easily interfaced with other measuring systems, have wide applicability, allow real‐time visualization, simple analytics for functional inference, large brain coverage, and be safe and cheap. These are some of the attributes that made fNIRS very attractive to neuroscientists, and any new fNIRS technology should retain these features.

## Why use fNIRS in cognitive neuroscience?

The field of cognitive neuroscience attempts to understand the mechanisms of cognition in the brain and to link this to our everyday lives in health, disease, and over the life span. Now that the field has matured from the point where any fMRI study was novel and most basic cognitive processes have been investigated, researchers are turning to new methods to understand how the brain allows us to function in the world.

One approach is the development of big data neuroimaging projects, such as the WU‐Minn Human Connectome Project, where over 1000 participants have been scanned over a period of approximately 4 h each (for an overview see Ref. 91), or the UK Biobank enterprise,^92^ where approximately 100,000 participants are being scanned. These large‐scale enterprises are remarkable undertakings with huge promise, although they face considerable challenges (see, e.g., Ref. 93). However, arguably a principal utility is in that they are one way of responding to a central criticism that could be made of the neuroimaging enterprise since the early 1990s. This is that there is a question of the degree to which neuroimaging findings help us understand how the brain enables us to cope with our everyday lives, since measurements are not taken *in situ*. An fMRI or PET study, for instance, places the participant in environment very far removed from normal everyday life, with severe restrictions on normal movement, hearing, etc. and often the tasks that the participant is asked to perform are unlike any that the participant would normally choose or be required to do in real life. Thus, the ecological validity of the results is uncertain. This is not a trivial matter if one wishes to project the scientific findings to, for example, addressing disability that cannot be measured in the scanner environment or to certain psychiatric or psychological problems. If the data have not been collected under the same conditions as the problems are demonstrated, then naturally one can be less certain about their relevance to understanding them, and the possibility of mediator variables and epiphenomena arise.

Fields related to cognitive neuroscience have been grappling with this issue for some time (e.g., Ref. 94). Big data neuroimaging enterprises may address this issue by collecting enough data to make statistical associations between real‐world variables and neuroimaging data, following an individual differences approach. However, within the field of neuropsychology, an additional approach has been developed to deal with this issue. This is the development of naturalistic methods of assessment, which stress the brain systems of interest *in situ*. In a neuropsychological context, this means using tasks that mimic the everyday situations where the participants demonstrate their difficulties (see, e.g., Ref. 95). This approach does not replace the use of experimental paradigms, but rather is a strong adjunct, and indeed is sometimes used as a validation of them. Recent developments in the technology of fNIRS means that neuroimaging can now follow a similar path, that is, stressing brain systems relevant to performance in everyday situations in order to gather data that are of more direct (rather than inferred) relevance. This is of relatively minor concern to some cognitive neuroscience researchers, but is fundamental to others. For those cognitive neuroscientists who study relatively basic sensory processes (such as the early stages of vision or audition), or where the cognitive resources are assumed to be relatively encapsulated (in the sense of Moscovitch and Umilta,^96^ i.e., impervious to top‐down control and dedicated to a particular activity), and the processes of interest are *not* related to any of the following: (1) bodily movement, including moving the head; (2) direct interpersonal interaction; (3) naturalistic behavior; (4) processing naturalistic stimuli (as opposed to stimuli presented on a computer display); (5) choosing how to respond, for example, speaking versus signaling with a hand; (6) physically moving around an environment; (7) situations involving a quiet environment; (8) situations involving being in an open environment; (9) any behavior not normally conducted while lying down, and largely immobile; and (10) any mental process or state of mind that is affected by being observed directly, then fMRI is well suited to its study. However, this is a long list of exclusionary conditions, and there are very many behaviors, situations, and mental processes which cannot currently be examined by fMRI. Indeed, it is perhaps the case that the neuroimaging community has become so used to the constraints that fMRI places upon experimentation that the lack of study of brain–behavior relations that correlate to, for example, free bodily movement or engaging in social interaction, etc. is rarely considered. Yet these are very substantial areas of cognition that are critical to understanding how the brain facilitates behavior in the real world, as well as understanding disorders of cognition that are often easier to measure in everyday situations than typical experimental ones (for examples, see Ref. 97).

## Overview of novel applications of fNIRS

**Figure 7:**
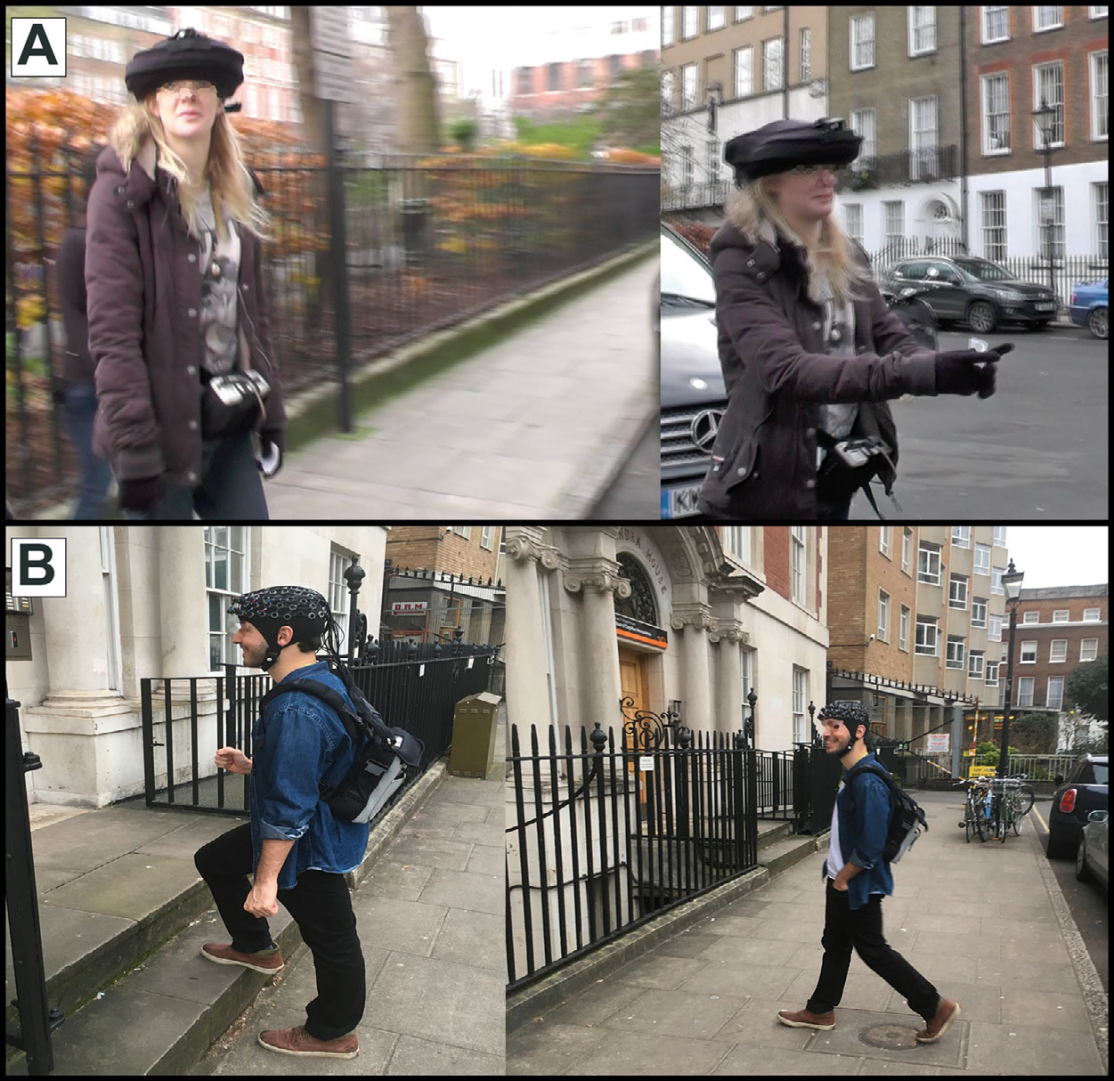
(A) Example of a participant carrying out the ecological prospective memory task described in Ref. 60 in the real world where brain activity is monitored over the PFC by a portable, wearable, and fibreless fNIRS system (WOT‐100, Hitachi, Japan; now sold by NeU Corporation, Japan). (B) Example of a participant freely moving in unrestrained situations outside the lab while functional brain activity is measured over the PFC through a portable and wearable fNIRS device (LIGHTNIRS, Shimadzu, Japan); such system is equipped with optical fibers that are connected to a control unit, carried through a backpack.

Recent technological advances in fNIRS present the opportunity to study the relations between brain activity and cognition in virtually all of the situations (1–10) listed above, in addition to those that one normally studies with fMRI. Following the development of the novel wearable and portable fNIRS devices, the past few years have seen the first studies investigating the feasibility of using fNIRS with freely moving participants. These studies have been conducted on healthy adults^43^, ^98^, ^99^, ^100^, ^101^, ^102^, ^103^, ^106^ and on patients with neurological deficits (e.g., Parkinson's disease^13^, ^100^ and mild cognitive impairments^14^), and demonstrated the ability of fNIRS of measuring brain hemodynamics and oxygenation in response to cognitive tasks performed while they are moving freely. It is even more exciting to see that these investigations can be performed outside the laboratory and in everyday life situations, as shown by the works in Refs. 60, 99, 101, and 102. In Figure 7, we provide examples of participants wearing portable and wearable fNIRS systems, showing how fNIRS allows to freely move in the environment and to accomplish the task with minimal restraints. Figure 7A refers to the study of Pinti *et al*.^60^ using the WOT‐100 fNIRS device (Hitachi, Japan; now sold by NeU Corporation, Japan) and Figure 7B shows the LIGHTNIRS^TM^ system (Shimadzu, Japan).

If wearable fNIRS devices are lightweight, compact, more robust to motion artifacts—especially the fibreless ones—than the conventional fNIRS instruments, and are to be used in naturalistic environments, detectors saturation due to the stray sunlight should be considered when using these new systems outdoor. This problem can be minimized by using shading caps (e.g., Fig. 7A), devices incorporating optical detectors with a high dynamic range or systems that include a reference detector with the aim of measuring the stray light only and subtracting it from the other detectors’ signals (e.g., as implemented in the Brite23^TM^ and OctaMon^TM^ developed by Artinis, the Netherlands).

**Figure 8:**
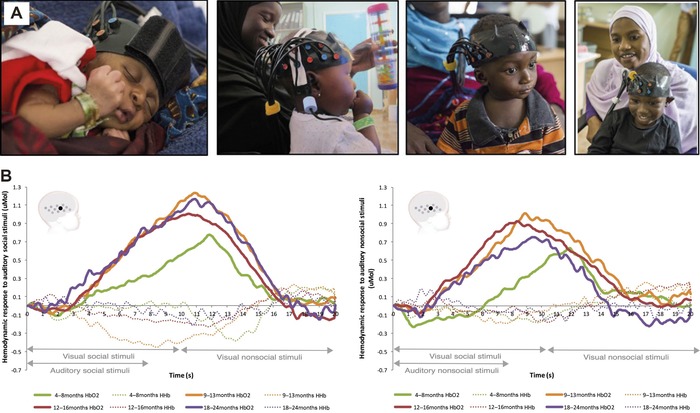
Examples of Gambian infants from 6 to 24 months undertaking the study described in Ref. 105 (A). Data were recorded using the UCL optical topography system with 12 channels. A change in the specialization to auditory social stimuli were found between the 4‐ and 8‐month‐old (green) infants and the 9–13 (orange), 12–16 (red), 18–24 (purple) months old cohorts in the anterior temporal cortex (B). HbO_2_ and HbR (here HHb) responses are indicated by full and dashed lines, respectively.^106^ The figure was modified with permission from Ref. 84 under the terms of the Creative Commons Attribution License (CC BY); photo credit to the Bill and Melinda Gates Foundation.

Other novel and promising applications of fNIRS concern the study of the effect of malnutrition and social or environmental difficulties on the neurodevelopment of infants living in low‐resource contexts. For instance, this is currently investigated by the BRIGHT project team^104^ on African infants. The first results on the cortical mapping of cognitive functions in Gambian infants^105^ (Fig. 8A) showed that fNIRS is able to provide objective markers and robust developmental curves, and, thanks to its low cost and portability, has the potential to be used in low‐resource settings. Figure 8B shows an example from the study in Ref. 105, where a change in specialization to auditory social cues was found in the anterior temporal cortex at 9 months of age.

Below, we present an overview of the current and potential applications of fNIRS in those fields of cognitive neuroscience that we think would benefit the most by the use of this novel neuroimaging modality. In particular, we focus on neuroimaging in natural situations and in social interaction, highlighting the current state of the field and potential future directions.

### The study of cognition in naturalistic situations: progress, challenges, and future directions

The possibility of monitoring brain activity in naturalistic contexts is especially important for those who study the PFC, since this exceptionally large region of the brain is thought to be intimately involved with voluntary behavior and dealing with open‐ended situations,^107^ and PFC activity measured by fNIRS has been found to be higher in realistic versus simulated everyday tasks (e.g., apple peeling^108^). Indeed, fNIRS lends itself well to studies of PFC activations, as Masataka *et al*.^109^ point out. There are two particular reasons for this. The first is technical: there is less hair at the front of the skull. But the second, far more important factor is the one alluded to above: large portions of PFC support mental processes involved with dealing with voluntary, self‐initiated behavior, especially in response to “open‐ended” or “ill‐structured” situations.^110^, ^111^

It has been argued for several decades that trying to measure frontal lobe function by placing people in highly constrained situations (environmentally, socially, and in terms of instruction and the possibilities for responding) risks a marked loss of construct validity (i.e., the degree to which you measure what you intend to measure; see Refs. 95 and 97 for reviews). The most common explanation is that since part of the role of PFC is to set the boundaries for behavior and responding through establishing various forms of top‐down control (e.g., “sculpting the response space”^112^), if the experimenter artificially provides these boundaries by placing the participant in a situation where only limited ways of moving, behaving, responding, or thinking are possible, this effectively eliminates much of the PFC‐supported mental processing that would normally be required in naturalistic or real‐world situations. In this way, there is good reason to suppose that certain mental processes supported by PFC that are highly predictive of competence in the real world are best investigated using naturalistic situations which attempt to introduce as few artificial (i.e., not commonly occurring outside the lab) constraints as possible. This issue of construct validity intersects neatly with that of *ecological validity*, first coined by Brunswik^113^ but has come to mean something rather different than he intended. These days it is most commonly used to refer to issues of generalizability (i.e., the degree to which the results of an experiment would hold across a range of situations outside it) and representativeness (the extent to which an experimental task corresponds in form and context to a situation encountered outside the laboratory).^95^ It is generally assumed that increases in the latter are a way to improve the former, and these are of particular concern where experimental findings are intended to illuminate, for example, clinical or ergonomic practice.

This naturalistic approach when used with fNIRS is already showing pleasing support for some principles of PFC function. For instance, in the study in Ref. 114, the authors used fNIRS equipment in a vehicle that was being driven on an expressway that was not open to the public. PFC activity was measured when the driver was either parked, accelerating, decelerating, maintaining a constant speed, or performing a U‐turn. The gateway hypothesis of supervisory attentional system model of PFC function (e.g., Ref. 115) would predict that activation while performing a U‐turn should be greater in rostral PC (BA 10) than during parking (since U‐turns are rarer and often more hazardous activities), and this is indeed what Yoshino *et al*.^114^ discovered. Similarly, the changes in patterns of activation over time as found in Ref. 116 during a gambling task are probably consistent with several theories of PFC function (e.g., Refs. 117 and 118). In this way, the findings from fNIRS investigations of PFC in naturalistic situations are useful tests of the extent to which we understand PFC function and organization at a theoretical level, as well as providing potentially useful data in terms of potential clinical, neuroergonomic, or engineering possibilities. Similarly, and overlapping with the study of PFC function, is the study of social interactions, discussed below, and the study of cortical activity during complex physical movement (e.g., Ref. 79). fNIRS promises measurements of enhanced ecological validity in these domains as well.

However, when studying behavior in naturalistic situations, new conceptual problems are created and should be considered. For instance, participants’ behavior may be voluntary and self‐generated in more ecologically valid contexts. This can make it more difficult to determine the time course of mental activity. The time at which a mental event occurs can be established with a high degree of confidence in some behavior al paradigms (e.g., simple stimulus–response tasks, in which mental events related to each stimulus, can be assumed to occur close to the time at which each stimulus is presented). However, in other situations it is far from straightforward to determine the temporal profile of mental activity putatively linked with a particular behavior. Overt behavior may be influenced by mental activity that has occurred considerably earlier. One methodological approach that has been adopted to investigate such situations it to average brain activity over multiple trials, time‐locked to a particular behavioral outcome, and search for the earliest point in time prior to this outcome that a difference in brain activity versus baseline can be detected. EEG studies investigating the readiness potential^119^ would be an example of this approach. A related approach is to sort trials into two or more categories (e.g., left versus right button presses) and search for the earliest time point prior to overt behavior at which brain activity distinguishes the two categories. The lateralized readiness potential is an example of this.^120^, ^121^ More recent studies have adapted this approach for fMRI.^122^

So, traditional neuroimaging methods such as fMRI are challenged by the study of self‐initiated or stimulus‐independent thought, which are central to an understanding of several fields within cognitive neuroscience (e.g., PFC function, social interaction, creativity, self‐initiated adaption to novel situations, etc.). How then might fNIRS assist in these investigations? The first advantage of fNIRS in this respect is of course the better temporal resolution compared to fMRI (although not better than EEG/MEG). This matters because the mental experiences under consideration (e.g., moments of insight) can be of relatively short duration. However, possibly an even more serious issue from an experimental standpoint is that the solutions to the complex problems that are typically used to provoke them take too long for these kinds of stimuli to be used in fMRI experiments.^123^ Here, fNIRS also provides a potential solution since relatively long periods of data acquisition can be tolerated much more easily by participants (e.g., well tolerated up to 1.5 h in case of the study in Ref. 60, where the fNIRS headset's weight was 700 g and the processing unit worn around the waist was 650 g), partly because they are free to move, but also of course because fNIRS is silent, and people of all heights and weights can be made to be comfortable (unlike fMRI). Linked to this is the ability to use automatic detection procedures such as AIDE^61^ described above to investigate the relationship between hemodynamic changes and behaviors or mental experiences of interest. In this way, stimulus‐independent thought (or cognition that is linked to, but distant from, a behavior) can be explored. This advantage is still made stronger by the quietness and lack of physical constraint, because unless the investigator is particularly interested in responses to, for example, the noise of a MRI scanner or the discomfort or stimuli provided by being immobilized, these are potential serious confounds since they may interfere greatly with the natural frequency or type of stimulus—independent thoughts. Incidentally, for this reason, fNIRS may be particularly useful for resting‐state data acquisition. Indeed, it is not known to what degree the results of resting‐state fMRI studies have been affected by these confounds: Are we really *resting* when we are immobilized and placed in a noisy and intimidating environment like an MRI scanner? fNIRS may be one method for finding out.

### Using fNIRS to explore social neuroscience

Neuroimaging studies of human social cognition have revealed a complex network of interacting brain regions with roles in social perception, emotion, imitation, and understanding of other people's mental states.^124^, ^125^ However, lying on your back in a dark noisy fMRI scanner is not a typical social situation and there are many aspects of a social interaction, which are hard to manipulate in these circumstances. These include the production of natural social behaviors (including posture, gesture, spontaneous mimicry, and unconstrained speech), the feeling of being watched by another person, and the continuous dynamic interaction between two people which characterizes natural conversation. This last factor in particular is gaining in importance, as researchers recognize that second person neuroscience, which studies real‐time interactive behavior, may be an important way to understand the social brain and psychiatric disorders.^126^

As detailed above, fNIRS can record brain activity in natural contexts and thus is compatible with all these richer social behaviors. In recent years, researchers have begun to use both single‐participant fNIRS in social contexts and to record from two or more participants simultaneously, giving new insights into the neural mechanism of social interaction. Here, we review some recent studies in this area, before highlighting challenges for the area and potential future directions.

#### Social neuroscience with fNIRS

A range of social perception and interaction studies in children and adults have been implemented in fNIRS. Some of these are similar to typical fMRI studies, examining face perception,^127^ emotion perception,^128^ and theory of mind^129^ in both child and adult populations. For example, the study in Ref. 130 showed that both infants and mothers have a positive fNIRS response in the PFC when seeing videos of the other smiling. Others take advantage of the flexibility of fNIRS to examine neural response to affective touch^131^ or to imitation behavior.^132^ For example, in the latter paper, participants completed an interactive task in which they saw a demonstrator make typical straight actions or abnormal curved actions and then had the opportunity to imitate them; right inferior parietal cortex showed a stronger activation signal when viewing the curved actions, which replicates previous fMRI studies. However, in fMRI, it is very hard to implement the imitation actions, and fNIRS provides more flexibility to explore the neural mechanisms of interactive behavior. There are even attempts to use fNIRS as a biofeedback mechanism for creating social virtual characters who can interact with a participant.^133^

Building on the participant‐friendly nature of fNIRS, a large number of studies use this method to explore the development of cognition and social cognition in infants, children, and individuals with developmental disorders. Such work tracks the development of body perception,^134^ response to direct gaze,^135^, ^136^ responses to speech^137^ and many other tasks. Reviewing all these papers is beyond the scope of the present article (see Refs. 35 and 138), so we highlight here a few developments. First, fNIRS has been used to identify differences in neural responses to social stimuli in 4‐month‐old infants at risk for autism,^139^ indicating a potential for early diagnosis. Similarly, adults with autism show differences in fNIRS responses when completing an imitation task^140^ and face viewing tasks.^141^ The flexibility of fNIRS to collect data in a wide range of contexts is also demonstrated by a project that tracks the development of malnourished infants in the Gambia, using fNIRS in rural conditions.^142^

#### Hyperscanning with fNIRS

Beyond the single‐participant studies reviewed above, one of the most exciting and rapidly growing areas of fNIRS is hyperscanning, where signals are recorded from two or more participants simultaneously. Hyperscanning in fMRI has been developed by chaining scanners together to record interactions in competitive/cooperative games,^143^, ^144^ sequential scanning of intimate partners that express emotional facial expressions,^145^ and sequential scanning of dyads who tell and listen to stories,^146^ However, critical social information that comes from face‐to‐face, real‐time updating of information about inner states, affect, and intentions is not well represented by these approaches. Using fNIRS hyperscanning, it is possible to both monitor brain activation and permit natural interaction behaviors. The first study of this form, published in 2012, examined neural activation in the frontal cortex of two participants playing a computer‐based cooperation game.^147^ A variety of methods are available to analyze these types of data, with a focus on measures of interbrain synchrony. This is typically quantified in terms of wavelet coherence, which provides an estimate of functional hyperconnectivity or Granger causality as a measure of effective hyperconnectivity.^148^ In both cases, a key analysis is to compare synchrony measures in true interactions to pseudointeractions created by shuffling data between participants or time‐windows while retaining the same measurement parameters. This provides an important baseline for statistical comparison. However, even this method cannot entirely remove the possibility that apparent synchrony between brains is driven by the two participants responding to a common input^146^ or even synchronization of physiological markers such as heartbeat and breathing. Developing new analysis methods to identify and isolate the causes of interbrain synchronization will be an important future direction in this area.

Despite these caveats, there have been a number of important advances since 2012. Studies have shown that it is possible to record and analyze fNIRS hyperscanning data in the context of singing,^149^ cooperative manual games,^150^ card games involving deception,^151^, ^152^ and imitation.^153^, ^154^ There are also studies that have gone beyond two‐person hyperscanning to record signals from three or four participants simultaneously. A study of three‐way conversation found that one person in each group of participants tended to act as a leader, and that the leader's temporoparietal junction showed greater synchronization of neural activation signals with the equivalent area in the follower participants, compared to the follower's synchronization with each other.^155^ This three‐way design is also able to overcome some of the problems of common input as all three participants are exposed to the same stimuli. Similarly, a four‐person hyperscanning has been attempted to explore different analysis options.^156^

**Figure 9:**
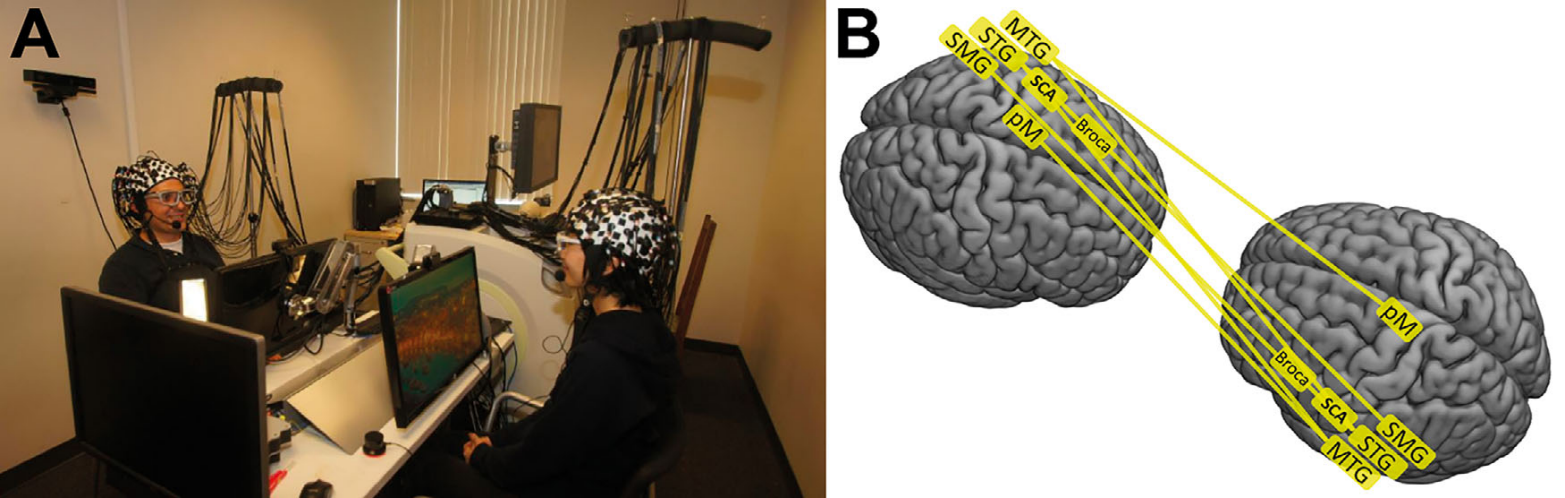
(A) The hyperscanning setup used in Ref. 81 is illustrated using the Shimadzu LABNIRS system (84 channels) synchronized with visual simulation monitors, eye‐tracking glasses, voice recording microphones, rotating dials providing a continuous analogue report of subjective responses, and wall mounted Kinect cameras for facial classifications. All components are the same for both participants and synchronized by triggers. Photo courtesy of Hirsch Brain Function Laboratory, Yale School of Medicine. (B) Cross‐brain synchrony is measured by wavelet coherence analysis and shown here for the condition of real eye‐to‐eye contact as compared to mutual gaze at a picture face and eyes. The illustration shows subsystems within the left temporal–parietal complex including the supramarginal gyrus (SMG), superior temporal gyrus (STG), middle temporal gyrus (MTG), the sub central area (SCA), and left premotor (pM) cortex that resonate more during real face‐to‐face eye‐to‐eye contact than viewing a face/eye picture (*P* **<** 0.001). These correlations between real partners disappeared when the partners were computationally scrambled, confirming that the coherence across partner brains was a result of actual real‐time reciprocal events and not general viewing of a moving face.^81^

To illustrate the value of fNIRS hyperscanning in more detail, we describe in depth the study in Ref. 81, which combines fNIRS with face and gaze tracking to explore the neural mechanisms for eye contact between two people. In this study, two participants completed trials where they gazed directly at each other's eyes or at a static photo of a face. fNIRS data were acquired with the Shimadzu LABNIRS^TM^ system with 134 channels equally divided between two participants. These acquisitions are synchronized with visual stimulus presentations, eye tracking, voice recording, Kinect^TM^ cameras for facial classifications, and response dials indicating continuous subjective ratings during the experiments, as shown in Figure 9A.

This illustrates the importance of combining the fNIRS itself with high‐resolution recordings of behavior to obtain the best data. This study used both GLMs to analyze data within subject and wavelet coherence measures (Fig. 9B) to analyze interbrain synchronization. In both measures, eye contact engages active neural systems associated with social engagement, which were not seen in the static picture condition. Furthermore, the subcentral gyrus (BA43), a previously undescribed functional region, was selectively activated and functionally connected to face, language, sensorimotor, and executive systems during eye‐to‐eye contact, and not during the noninteractive condition, suggesting a foundational mechanism for continuous, bidirectional streaming of social interactions. These findings contribute to an emerging model for human‐to‐human interactions and suggest that a subset of neural operations is mutually engaged during live eye‐to‐eye contact. Observations of cross‐brain coherence such as these (Fig. 9B) introduce novel opportunities to understand rapid sensory processes dedicated to interpersonal interaction not previously investigated due, in part, to conventional block designs, sparse temporal sampling, and restrictions on imaging during natural two‐person interactions. Future fNIRS research will focus on the sensory processes engaged during coherent neural activity during live and spontaneous interactions as a key sensory element that drives neural responses during real person‐to‐person interactions.

#### Future directions within social neurosciences and hyperscanning

To summarize, fNIRS for the study of human social interaction is a rapidly growing field with excellent potential. When used in conjunction with careful recording of behavior and in hyperscanning contexts, fNIRS has the potential to reveal important facets of the brain mechanisms of interaction, which cannot be captured in the fMRI environment. In the ongoing quest to build a field of second person neuroscience^157^ and to test the interactive brain hypothesis,^158^ fNIRS hyperscanning will be an essential tool.

## The future of fNIRS

fNIRS occupies a significant and rapidly expanding space within neuroscience, providing huge neuroimaging capacity with fewer constraints upon participants’ behavior. In fact, fNIRS allows the neuromonitoring of a wide range of populations, from newborns to the elderly. More interestingly, this can now be performed in more realistic environments and in everyday lives, thanks to the advances in hardware development. The availability of wearable fNIRS devices, for example, paves the way for new and potentially revolutionary neuroimaging investigations that might grow exponentially over the next years, particularly in the domains of real‐world cognition, social interaction, and neurodevelopment.

However, these are still very early days for fNIRS in cognitive neuroscience, yet there is little doubt that the next few years will see new ways of applying the methodology. For instance, the dynamics of the relationship between oxyhemoglobin and deoxyhemoglobin signals in different brain regions and across different tasks and conditions remains largely to be investigated, and there may be fundamental discoveries in these kinds of investigations. In addition, fNIRS instrumentation can also be easily interfaced with other physiological measurements, including other neuroimaging modalities (such as EEG or fMRI^52^), systemic measurements (such as heart rate, blood pressure, breathing rate, etc.), and behavior (such as eye tracking, motion capture, video recordings, etc.). This will allow a 360° view of how neurodynamics are coordinated with other changes within the body, which could be fundamental to work on, for example, anxiety, stress, or similar work at the interface between emotion and cognition. Similarly, the improved sampling rate of fNIRS over fMRI may offer approaches and considerations that we have not considered yet including new connectivity analyses. In many ways, the early days of fNIRS mirror the early days of fMRI with respect to scientific development. fNIRS physicists, engineers and methods community are investigating what is possible with the new method and, to an degree, this is occurring independently of scientific questions that might drive them, as few cognitive neuroscientists know enough about fNIRS to have spent a lot of time considering what questions this new technique allows them to ask, which they cannot consider with the established methods of fMRI or MEG. During the early history of fMRI, a stage was soon reached where the equipment and methods were made accessible enough to those people interested in determining brain–behavior relations in humans that they could start thinking about what questions they could ask. This led to a huge explosion of interest, as the interaction between cognitive scientists and medical physicists became routine, and they started to share a common scientific goal, and interdisciplinary collaborations became the norm. fNIRS is now at that cusp too. We now know enough to have some idea of its potential as a method, and the equipment is sophisticated enough to be useful, and is easy (and cheap) enough to be accessible to a wide variety of cognitive neuroscientists. Indeed, the safety and cheapness have huge potential for its widespread use in a way beyond that possible with fMRI. Therefore, what is almost certain to happen now is an enormous increase in the use of fNIRS that will drive both new scientific questions, and new ways of addressing them that mirrors the transformational changes to the discipline that happened with fMRI. This will be stimulating for *early adopters* as new studies that have never been done before due to limitations of previous technologies can now be performed.

Indeed, the growth of fNIRS is likely to be faster than of fMRI because the scientific infrastructure (e.g., software packages for analysis, standard ways of designing experiments) now exists in a way that it did not before fMRI. Moreover, there are many neuroscientists who have until now been limited in their investigations by the constraints imposed by fMRI and other methods. Hence, there is a ready audience. These investigators are increasingly adopting fNIRS to overcome some of the issues related to other neuroimaging modalities, pushing the boundaries of the application of the technique, but also increasing the demand for new analytic methodologies and procedures that allow its effective use, often alongside the more established methods they know well. The future of cognitive and social neuroscience seems brighter with fNIRS.

## Competing interests

The authors declare no competing interests.
